# Bioinformatic Analysis Combined With Experimental Validation Reveals Novel Hub Genes and Pathways Associated With Focal Segmental Glomerulosclerosis

**DOI:** 10.3389/fmolb.2021.691966

**Published:** 2022-01-04

**Authors:** Yan-Pei Hou, Tian-Tian Diao, Zhi-Hui Xu, Xin-Yue Mao, Chang Wang, Bing Li

**Affiliations:** ^1^ Department of Nephrology, Second Affiliated Hospital of Harbin Medical University, Harbin, China; ^2^ Department of Pediatrics, First Affiliated Hospital of Harbin Medical University, Harbin, China; ^3^ Department of Nephrology, Second Affiliated Hospital of Hainan Medical University, Haikou, China

**Keywords:** bioinformatic analysis, FSGS, GEO, hub genes, pathway

## Abstract

**Background:** Focal segmental glomerulosclerosis (FSGS) is a type of nephrotic syndrome leading to end-stage renal disease, and this study aimed to explore the hub genes and pathways associated with FSGS to identify potential diagnostic and therapeutic targets.

**Methods:** We downloaded the microarray datasets GSE121233 and GSE129973 from the Gene Expression Omnibus (GEO) database. The datasets comprise 25 FSGS samples and 25 normal samples. The differential expression genes (DEGs) were identified using the R package “limma”. Gene Ontology (GO) function and Kyoto Encyclopedia of Genes and Genomes (KEGG) pathway enrichment analyses were performed using the database for Annotation, Visualization and Integrated Discovery (DAVID) to identify the pathways and functional annotation of the DEGs. The protein–protein interaction (PPI) was constructed based on the Search Tool for the Retrieval of Interacting Genes (STRING) database and visualized using Cytoscape software. The hub genes of the DEGs were then evaluated using the cytoHubba plugin of Cytoscape. The expression of the hub genes was validated by quantitative real-time polymerase chain reaction (qRT-PCR) using the FSGS rat model, and receiver operating characteristic (ROC) curve analysis was performed to validate the accuracy of these hub genes.

**Results:** A total of 45 DEGs including 18 upregulated and 27 downregulated DEGs, were identified in the two GSE datasets (GSE121233 and GSE129973). Among them, five hub genes with a high degree of connectivity were selected. From the PPI network, of the top five hub genes, FN1 was upregulated, while ALB, EGF, TTR, and KNG1 were downregulated. The qRT-PCR analysis of FSGS rats confirmed that the expression of FN1 was upregulated and that of EGF and TTR was downregulated. The ROC analysis indicated that FN1, EGF, and TTR showed considerable diagnostic efficiency for FSGS.

**Conclusion:** Three novel FSGS-specific genes were identified through bioinformatic analysis combined with experimental validation, which may promote our understanding of the molecular underpinning of FSGS and provide potential therapeutic targets for the clinical management.

## Introduction

Focal segmental glomerulosclerosis (FSGS) is the most common form of glomerular disease that leads to end-stage renal disease (ESRD) in America (Rosenberg and Kopp, 2017) and is the fourth leading cause in China (Xu et al., 2016). The incidence and prevalence of FSGS varies among different regions and ethnicities: males are 1.5–2 times more likely than females to develop FSGS, and blacks are 4 times more likely than whites to develop this disease (Kitiyakara et al., 2003). FSGS accounts for nearly 40% of adult nephrotic syndrome (D’Agati et al., 2011), and approximately 50% of patients will progress to ESRD within 3–8 years after the diagnosis of FSGS (Podestà and Ponticelli, 2020). The pathological features of FSGS involve lessened podocyte foot process, demise of podocytes and bareness of the glomerular basement membrane, leakage of nonspecific plasma proteins, capillary collapse, mesangial matrix dilation, infiltration of foam cells in capillaries, hyaluronic degeneration and epithelial proliferation (Campbell and Tumlin, 2018). FSGS is a complex disease with multiple clinicopathological features, the mechanisms of which remain less understand (Podestà and Ponticelli, 2020). Currently, the treatment of FSGS relies on the long-term use of steroids and immunosuppressants, both of which cause serious side effects (Bose and Cattran, 2014). Apart from that, a majority of FSGS patients often cannot be diagnosed at early stages of the disease due to the absence of valid diagnostic strategies (Wiesława et al., 2012). Therefore, understanding the exact molecular mechanism of FSGS may facilitate the effective diagnosis and treatment strategies.

Recently, bioinformatics has become a powerful tool to provide a comprehensive and in-depth understanding of the molecular mechanisms of disease to identify potential biomarkers and therapeutic targets (Zhou et al., 2018). Many bioinformatics analysis methods can be implemented based on the gene expression profiles, such as differential expression genes (DEGs) studies, Venn diagrams, functional and pathway enrichment studies, and protein-protein interaction (PPI) network analysis (Shu et al., 2018). R is a software and operating environment for statistical analysis and data visualization (Sugiyama et al., 2018). A wide variety of R packages, such as Pheatmap, Volcano Map and Venn Diagram, which can be used to implement bioinformatics analysis functions. Microarray technology and bioinformatics analysis can screen the genomes and transcriptome of FSGS samples and normal samples and distinguish DEGs. Genetic analysis data from patients with FSGS have been uploaded to the Gene Expression Omnibus (GEO) database but have not been analyzed. Therefore, we reanalyzed the FSGS microarray data from the GEO, which may provide insights for the diagnosis and treatment of patients with FSGS.

FSGS is a broad group of diseases with unique pathological mechanisms, and studies should attempt to identify new biomarkers. As a consequence, it is very important to study the molecular or pathological mechanisms of FSGS with the aim of establishing more effective methods of diagnosis and treatment.

## Materials and Methods

### Data Source

The gene expression profiles analyzed in this study were downloaded from the National Center for Biotechnology Information GEO database (https://www.ncbi.nlm.nih.gov/geo/). A total of 508 data points for FSGS were retrieved from the database. After elaborative screening, we selected two microarray datasets (GSE121233 and GSE129973) that fit our requirements, and both of these datasets are based on the GPL17586 platform [Affymetrix Human Transcriptome Array 2.0 (HTA-2_0)].

### Data Processing of DEGs

Microarray data is calibrated and normalized using the R software. DEGs (FSGS vs normal) were identified from the GSE121233 and GSE129973 datasets using the R package ‘limma’ (Gentleman et al., 2004). The DEGs that met the following criteria were considered significant DEGs: adjusted *p* < 0.05 and |logFC| ≥1. The identified DEGs were visualized using heatmaps and volcano plots. Venn Diagram (bioinformatics.psb.ugent.be/webtools/Venn/) was used to select genes with consistent alterations in expression in both datasets.

### Gene Ontology (GO) and Kyoto Encyclopedia of Genes and Genomes (KEGG) Enrichment Analyses of DEGs

GO and KEGG are databases of gene-related functions that are stored based on various taxonomic annotations (Gene Ontology Consortium, 2015). A GO enrichment analysis generally involves the following three categories: biological process (BP), molecular function (MF) and cell composition (CC). The KEGG database stores a wealth of data on genomes, biological pathways, diseases, chemicals and drugs. The database for Annotation, Visualization and Integrated Discovery (DAVID version 6.8, https://david. ncifc rf. gov/) is an online bioinformatics database that provides users with a complete set of annotated gene and protein function information for the extraction of biological information (Dennis et al., 2003). The DEGs were uploaded to DAVID for KEGG and GO analyses. A difference was considered statistically significant if *p* < 0.05.

### Construction of the PPI Network and Identification of Hub Genes

The Search Tool for the Retrieval of Interacting Genes (STRING, version 11.0, http://strin g-db. org/) database is designed to analyze PPI information (Szklarczyk et al., 2015). To evaluate the potential PPI relationships, the identified DEGs were mapped to the STRING database based on an interaction score of at least 0.15. Subsequently, Cytoscape software (version 3.8.1, www.cytoscape.org/) was employed to visualize the PPI network (Shannon et al., 2003). The node connectivity is directly proportional to the stability of the entire network. CytoHubba Version 0.1, a plugin in Cytoscape, was used to calculate the degree of each protein node (Saito et al., 2012). In our study, the top five genes were identified as hub genes.

### The Disease Portals

The Rat Genome database (RGD) disease Portals (http://rgd.mcw.edu/wg/portals/) provides researchers around the world with critical datasets and software tools to understand the mechanisms of diverse diseases in rats (Hayman et al., 2016), humans, and mice. We searched the term for FSGS in this disease portal.

### Experimental Animals

The animal study was reviewed and approved by the Animal Laboratory Committee of Harbin Medical University. Eight-week-old male Sprague-Dawley rats weighing 200–220 g were purchased from the laboratory of the Second Affiliated Hospital of Harbin Medical University. All rats were maintained in an air-conditioned room and provided unrestricted access to water and food (22 ± 2°C; 12:12 h light dark cycle). FSGS was induced by the two intravenous administrations of adriamycin (4 mg/kg on day 1 and 3 mg/kg on day 14; Solarbio, Beijing, P.R. C., n = 8) (Lee and Harris, 2011). The rats in the control group received an injection of normal saline through the tail vein (n = 8). The rats were euthanized under anesthesia (10% chloral hydrate by peritoneal injection), and all efforts were undertaken to minimize pain and discomfort. FSGS was confirmed 12 weeks later via renal pathology.

### Quantitative Real-Time Polymerase Chain Reaction (qRT-PCR)

Total RNA was extracted from rat kidneys using TRIzol reagent (Invitrogen Life Technologies, Carlsbad, CA, United States). Using high-capacity cDNA reverse transcription kit (Code No. FSQ-101, OSAKA, JAPAN) to reverse transcription of total RNA. The primer sequences for qRT-PCR were as follows: EGF primers 5-GAT​GTA​GGT​CAC​CCA​TTC​TCT​C-3 and 5-CAG​TCC​TCT​TGT​TCA​CCC​TTA​T-3; TTR primers 5-GCT​CAC​CAC​AGA​TGA​GAA​GTT-3 and 5-AAA​CCA​CCT​CTG​CGT​ATT​CAT -3; FN1 primers 5-CCA​AGT​ACA​TTC​TCA​GGT​GGA​G-3 and 5-GGT​CAG​GCC​TTT​GAT​GGT​ATA​G-3. For qRT-PCR, SYBR Green PCR Master Mix (Cat.NO.04913850001, Roche, Germany) was used according to the manufacturer’s instructions with a BIO-GENER Real-Time System (CHINA). Each sample was repeated three times. The experimental cycle threshold (CT) values were normalized to values obtained for glyceraldehyde-3-phosphate dehydrogenase (GAPDH) measured on the same plate, and the 2^−ΔΔCT^ method was used to determine the fold difference in gene expression (Schmittgen and Livak, 2008).

### Statistical Analysis

R software (version ×64 4.0.3), SPSS (version 25.0; IBM, Chicago, IL, United States) and GraphPad Prism 8.0 were used for all other statistical analyses and visualizaton. *p* < 0.05 was considered to indicate statistical significance. When the data fit the normal distribution, an independent two-sample *t* test was used in the comparison of means between groups. When the data did not conform to a normal distribution, the Mann–Whitney *U* test was used for unpaired continuous variables between groups. The diagnostic accuracy of selected genes was evaluated by receiver operating characteristic (ROC) curves and corresponding areas under the ROC curves (AUCs). First, the 25 groups of data of GSE121233 and GSE129973 are integrated, and then the Graphpad software was used to make ROC curve and evaluate AUC. An AUC of 0.6–0.7 was considered poor, 0.7–0.8 was considered moderate, 0.8–0.9 was considered good, and >0.9 was considered excellent.

## Results

### Identification of DEGs

**FIGURE 1:**
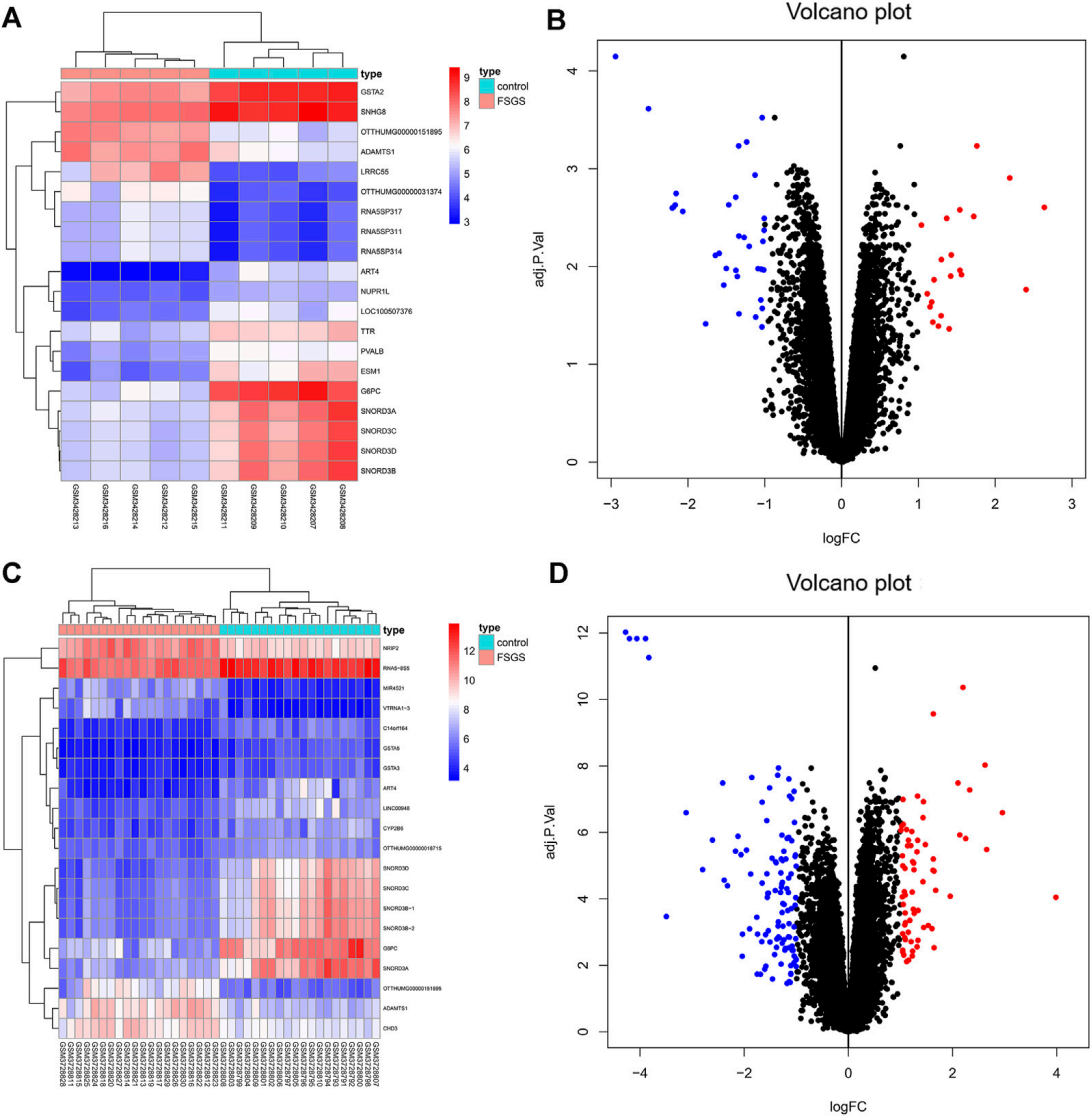
Heatmap **(A)** and volcano plot **(B)** of GSE121233 DEGs. Heatmap **(C)** and volcano plot **(D)** of GSE129973 DEGs. The heatmap visually shows the difference in gene expression between the FSGS group and the healthy control group. Each dot in the volcano map represents a gene. Blue represents downregulated genes, and red represents upregulated genes. Abbreviations: DEGs, differential expression genes.

**FIGURE 2:**
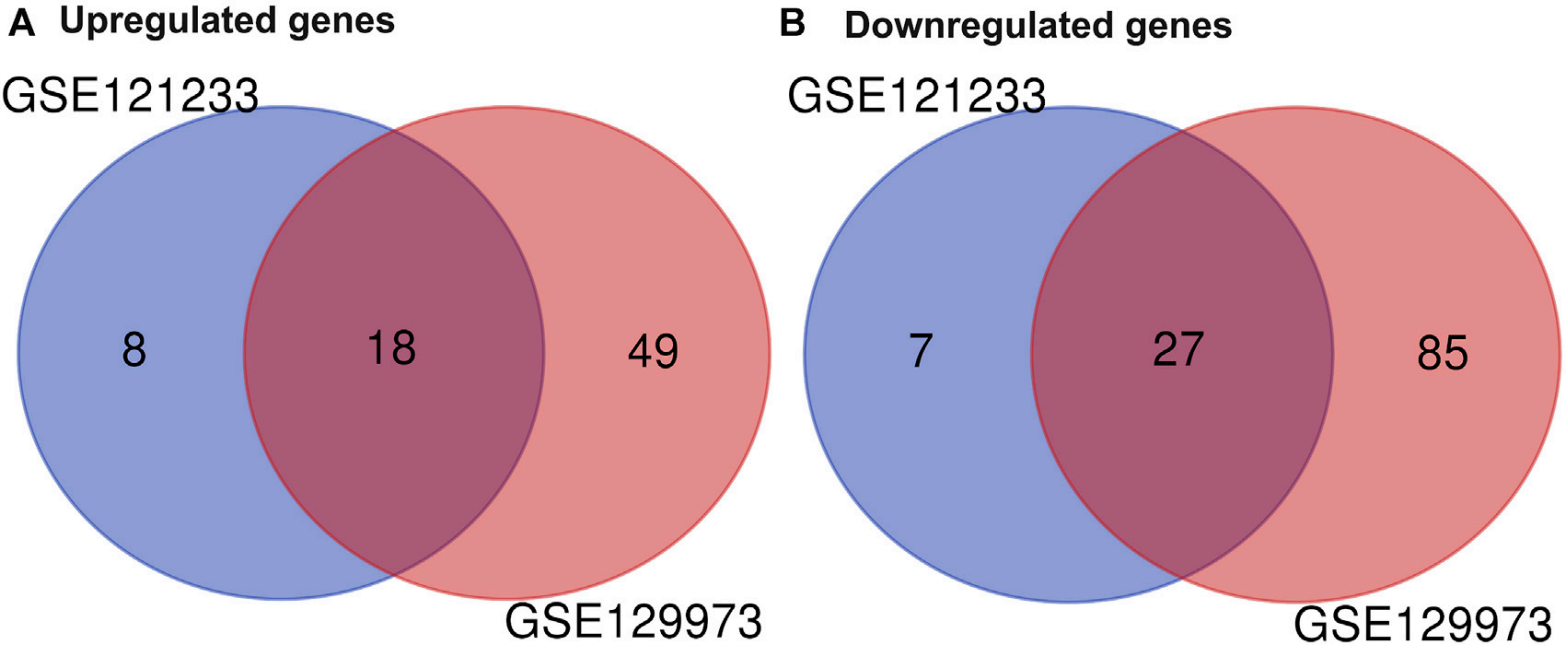
Venn diagram of DEGs common to two GEO datasets. A total of 45 consistently expressed genes were identified from GSE121233 and GSE129973, including 18 upregulated genes **(A)** and 27 downregulated genes **(B)** in FSGS kidney tissues compared to the control. Different color areas represented different datasets. The cross areas indicate the common expressed genes. Abbreviations: DEGs, differential expression genes; GEO, Gene Expression Omnibus.

Two datasets of gene expression profiles (GSE121233 and GSE129973) were selected in this study. GSE129973 contains 20 FSGS samples and 20 normal samples, and GSE121233 includes five FSGS samples and five normal samples. All DEGs were screened by comparing the FSGS samples with the normal samples. A total of 60 DEGs were identified from GSE121233 based on the criteria *p* < 0.05 and |logFC| ≥1, and these included 26 upregulated genes and 34 downregulated genes. In addition, 179 DEGs were identified from the gene expression dataset GSE129973, and these consisted of 67 upregulated genes and 112 downregulated genes. A heatmap of the DEGs obtained from GSE121233 and a volcano plot of this dataset are displayed in Figures 1A, B, and a heatmap of the DEGs obtained from GSE129973 and a volcano plot of this dataset are displayed in Figures 1C, D. A Venn diagram was then ploted to select the common DEGs in the two datasets (as shown in Figure 2). The results showed that the expression of 45 genes were significantly altered in both datasets, including 18 significantly upregulated DEGs and 27 significantly downregulated genes. In this way, we identified highly plausible genes with significant alterations in expression in FSGS.

### Functional Enrichment Analyses of DEGs

**FIGURE 3:**
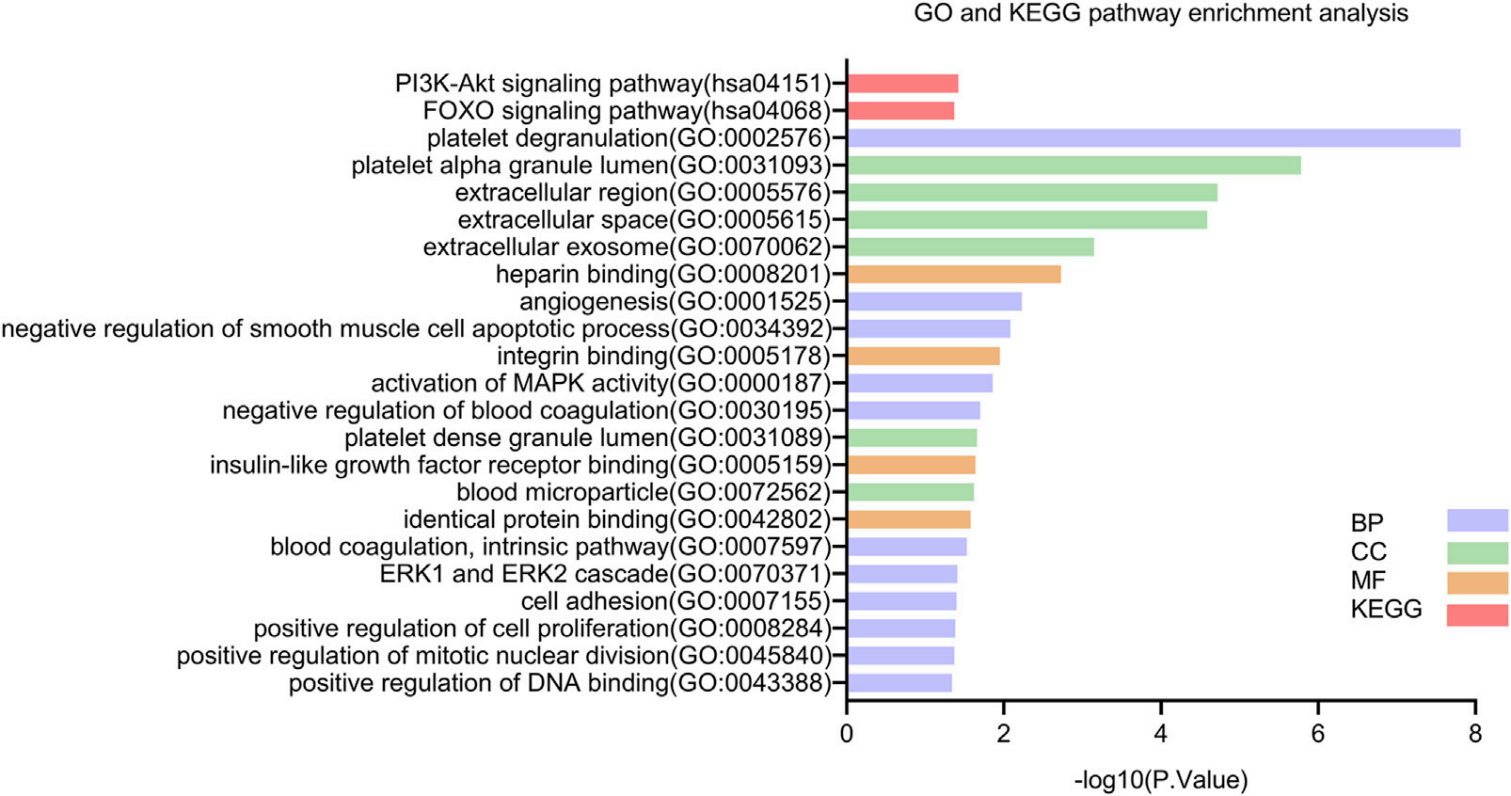
KEGG and GO enrichment analyses of DEGs. Abbreviations: BP, biological process; CC, cellular component; MF, molecular function; DEGs, differential expression genes; GO, Gene Ontology; KEGG, Kyoto Encyclopedia of Genes and Genomes.

Next, we explored the possible functions of these DEGs and the signaling pathways affected. The results from the GO function and KEGG pathway enrichment analyses of the DEGs using DAVID with the cutoff criterion of *p* < 0.05 are shown in Figure 3. The results from the GO analysis indicated that the DEGs were mainly enriched in BPs, including platelet degranulation, angiogenesis, cell adhesion, positive regulation of cell proliferation, activation of MAPK activity, negative regulation of smooth muscle cell apoptotic process, negative regulation of blood coagulation, blood coagulation, intrinsic pathway, ERK1 and ERK2 cascade, positive regulation of mitotic nuclear division, and positive regulation of DNA binding. In the CC category, the DEGs were enriched in platelet alpha granule lumen, extracellular region, extracellular space, extracellular exosome, platelet dense granule lumen, and blood microparticle. As for MF, the DEGs were significantly enriched in integrin binding, heparin binding, insulin-like growth factor receptor binding, and identical protein binding. In addition, the KEGG pathway analysis showed that the DEGs were mainly enriched in the PI3K-Akt signaling pathway and the FOXO signaling pathway.

### Construction of the PPI Network and Identification of Hub Genes

**FIGURE 4:**
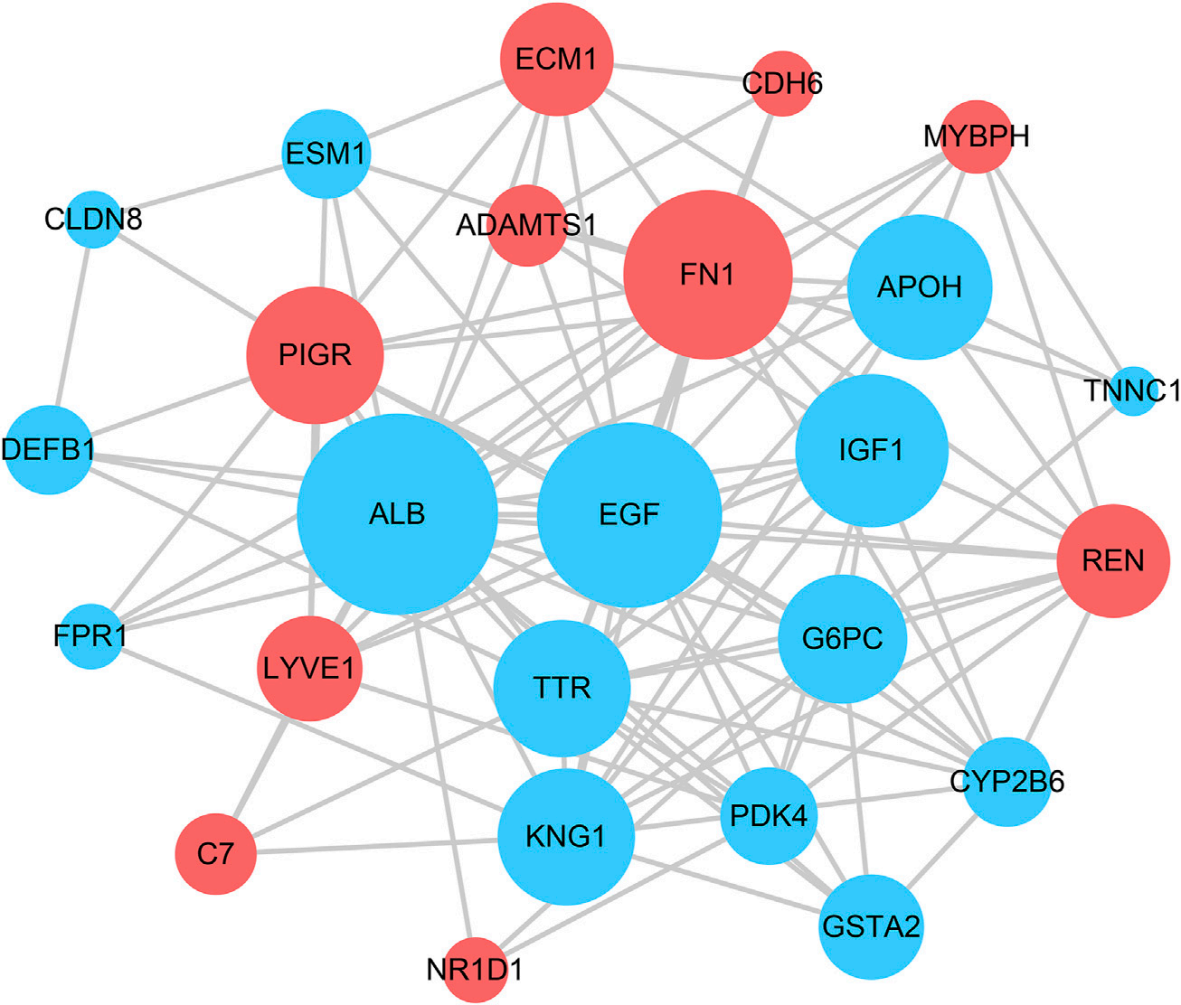
Protein–protein interaction network constructed with the differentially expressed genes. Red indicates that gene expression is upregulated; blue indicates that gene expression is downregulated.

The STRING tool was used to construct the PPI network based on the DEGs. The PPI network model was visualized using Cytoscape software (as shown in Figure 4). Each node represents a protein, and an edge represents an interaction between proteins. The size of the nodes is adjusted by the degree. The top five genes were identified using cytoHubba based on their connectivity in the PPI network (as shown in Table 1).

**TABLE 1:** Top five hub genes with a higher degree of connectivity.

Rank	Name	Description	Degree	Status
1	ALB	Albumin	21	down-regulated
2	EGF	Epidermal growth factor	19	down-regulated
3	FN1	Fibronectin 1	17	up-regulated
4	TTR	Transthyretin	14	down-regulated
5	KNG1	Kininogen 1	12	down-regulated

### DEGs Analysis Based on RGD Disease Portals

A search of the RGD disease Portals Genome database set along with FSGS confirmed that there are 51 human genes related to the etiology and prognosis of FSGS (as shown in Table 2). Among the 51 genes, G6PC and REN were the same as the screened genes.

**TABLE 2:** Fifty-one human genes related to the etiology and prognosis of FSGS.

Genes	Official full name
ACTN4	Actinin alpha 4
ANLN	Anillin actin binding protein
APOL1	Apolipoprotein L1
CD2AP	CD2 associated protein
CLCN5	Chloride voltage-gated channel 5
CLCNKB	Chloride voltage-gated channel Kb
COQ6	Coenzyme Q6, monooxygenase
COQ8B	Coenzyme Q8B
CRB2	Crumbs cell polarity complex component 2
G6PC	Glucose-6-phosphatase catalytic subunit
INF2	Inverted formin 2
ITGA3	Integrin subunit alpha 3
JAG1	Jagged canonical Notch ligand 1
MT-CO1	Mitochondrially encoded cytochrome c oxidase I
MT-CO2	Mitochondrially encoded cytochrome c oxidase II
MT-CO3	Mitochondrially encoded cytochrome c oxidase III
MT-ND1	Mitochondrially encoded NADH: ubiquinone oxidoreductase core subunit 1
MT-ND4	Mitochondrially encoded NADH: ubiquinone oxidoreductase core subunit 4
MT-ND5	Mitochondrially encoded NADH: ubiquinone oxidoreductase core subunit 5
MT-ND6	Mitochondrially encoded NADH: ubiquinone oxidoreductase core subunit 6
MT-TF	Mitochondrially encoded tRNA-Phe (UUU/C)
MT-TH	Mitochondrially encoded tRNA-His (CAU/C)
MT-TL1	Mitochondrially encoded tRNA-Leu (UUA/G) 1
MT-TQ	Mitochondrially encoded tRNA-Gln (CAA/G)
MT-TS1	Mitochondrially encoded tRNA-Ser (UCN) 1
MT-TS2	Mitochondrially encoded tRNA-Ser (AGU/C) 2
MT-TW	Mitochondrially encoded tRNA-Trp (UGA/G)
MYO1E	Myosin IE
NARS2	Asparaginyl-tRNA synthetase 2, mitochondrial
NPHS2	NPHS2 stomatin family member, podocin
NUP107	Nucleoporin 107
NUP133	Nucleoporin 133
NUP160	Nucleoporin 160
NUP205	Nucleoporin 205
NUP85	Nucleoporin 85
NUP93	Nucleoporin 93
PAX2	Paired box 2
PLCE1	Phospholipase C epsilon 1
PTPRO	Protein tyrosine phosphatase receptor type O
REN	Renin
SCARB2	Scavenger receptor class B member 2
SEC61A1	SEC61 translocon subunit alpha 1
SGPL1	Sphingosine-1-phosphate lyase 1
SLC12A3	Solute carrier family 12 member 3
SLC37A4	Solute carrier family 37 member 4
SMARCAL1	SWI/SNF related, Matrix associated, Actin dependent regulator of chromatin, Subfamily a like 1
TBC1D8B	TBC1 domain family member 8B
TRPC6	Transient receptor potential cation channel subfamily C member 6
VPS33A	VPS33A core subunit of CORVET and HOPS complexes
WDR73	WD repeat domain 73
WT1	WT1 transcription factor

### Validation of Hub Genes

**FIGURE 5:**
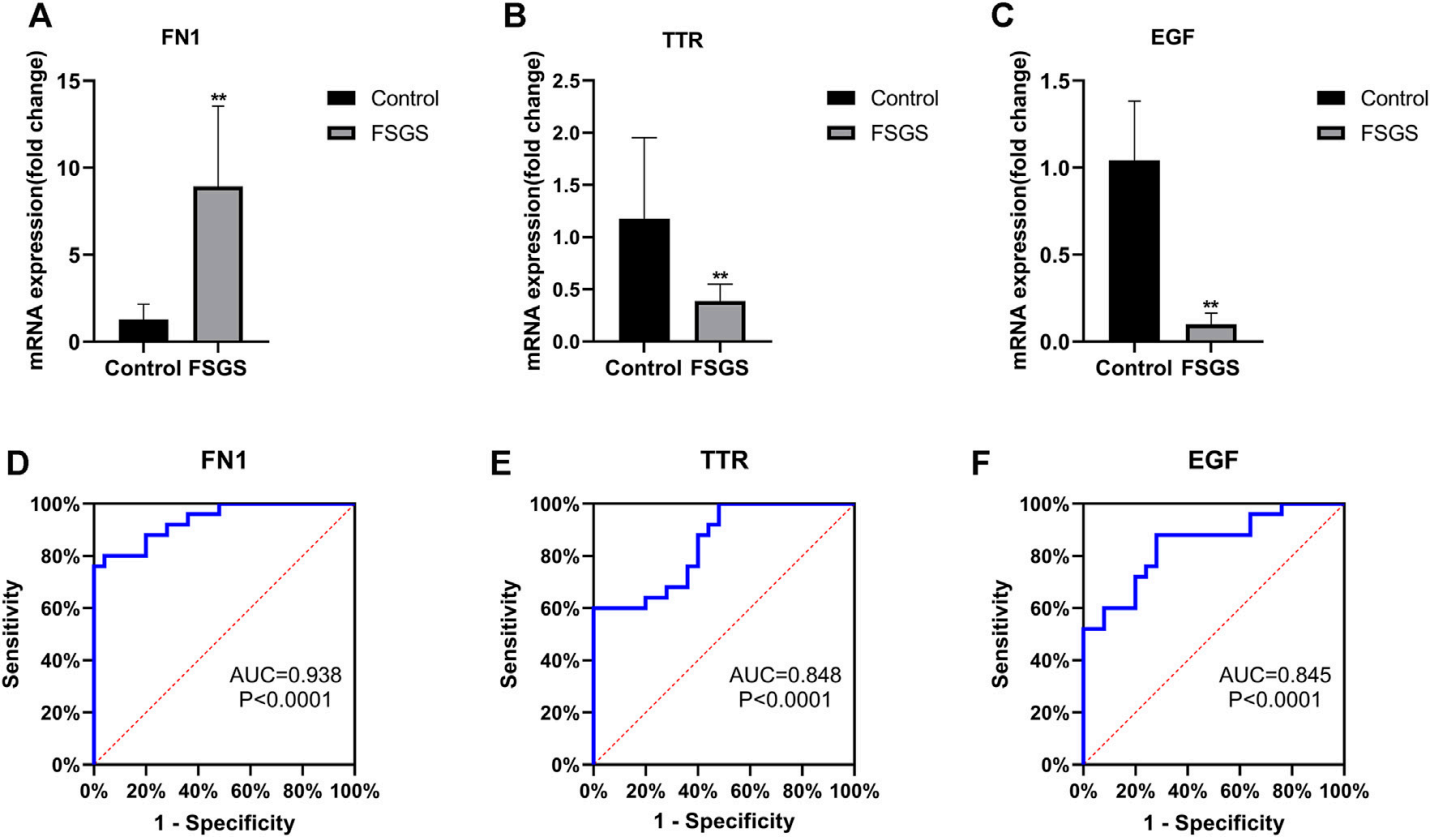
Quantitative real-time PCR of FN1 **(A)**, TTR **(B)** and EGF **(C)** in the FSGS rat model compared to the control. ROC curve analysis of FN1 **(D)**, TTR **(E)** and EGF **(F)** for predicting FSGS. Data are expressed as the mean ± SEM; **p < 0.01 vs the control group. The areas under the curves (AUCs) were 0.938 (95% CI: 0.876–1.000, *p* < 0.0001) for FN1 (D), 0.848 (95% CI: 0.744–0.952, *p* < 0.0001) for TTR **(E)**, and 0.845 (95% CI: 0.737–0.953, *p* < 0.0001) for EGF **(F)**. Abbreviations: CI, confidence interval; ROC, receiver operating characteristic.

To validate the results from the bioinformatics analyses, the expression of the hub genes in FSGS and normal kidney tissues was analyzed by qRT-PCR. In agreement with the bioinformatics results, FSGS tissues exhibited upregulated expression of FN1 (Figure 5A) and downregulated expression of TTR (Figure 5B) and EGF (Figure 5C) compared with normal tissues.

### Accuracy of the Candidate Genes in the Diagnosis of FSGS

A ROC curve analysis was performed to investigate the accuracy of these genes as biomarkers for FSGS. As shown in Figure 5, the AUCs were 0.938 (*p* < 0.0001) with a cutoff value of 5.935 (80% sensitivity, 96% specificity) for FN1 (Figure 5D), 0.848 (*p* < 0.0001) with a cutoff value of 6.496 (60% sensitivity, 100% specificity) for TTR (Figure 5E), and 0.845 (*p* < 0.0001) with a cutoff value of 5.756 (88% sensitivity, 72% specificity) for EGF (Figure 5F).

## Discussion

Primary FSGS has become one of the most common causes of adult idiopathic glomerular disease. Although the etiology of primary FSGS is thought to be due to circulating osmotic factors, the actual factors have not been identified (Savin et al., 1996). In recent years, bioinformatics has become a powerful tool for the high-throughput identification of potential biomarkers (Zhou et al., 2019). However, due to the huge amount of data produced by high-throughput technologies, a bioinformatics analysis of one microarray dataset could yield false-positive results, and we therefore selected two microarray datasets from the GEO database to screen for DEGs in FSGS and identify potential biomarkers.

We screened two gene expression microarray datasets from the GEO database that met our established requirements and performed GO and KEGG enrichment analyses as well as PPI analyses to clarify the molecular mechanism of the DEGs identified from the comparison of FSGS and healthy human kidney tissues. With the 25 FSGS samples and 25 normal samples from GSE121233 and GSE129973, 18 common upregulated genes and 27 common downregulated genes were screened. Next, we predicted DEGs functions based on GO and KEGG pathway annotations. For GO enrichment, the DEGs were considerably concentrated in platelet degranulation, extracellular exosome, extracellular region, extracellular space, and platelet alpha granule lumen. For the KEGG pathway, the DEGs were considerably concentrated in the PI3K/Akt signaling pathway and the FOXO signaling pathway. The PI3K/Akt pathway, as a key regulator of cell stress, has received extensive attention regarding its molecular mechanism and in the treatment of various diseases. The PI3K/Akt pathway is a very important intracellular signaling pathway that is associated with cell quiescence, cell proliferation, cancer and longevity (Xie et al., 2018). The FOXO signaling pathway is associated with cell differentiation, apoptosis, cell proliferation, DNA damage and repair, and oxidative stress (Mohd et al., 2017).

A PPI network, involving 30 nodes and 119 edges, was constructed to investigate the interrelationship of the DEGs. PPI network consists of highly-connected proteins and less-connected proteins, which makes a network tolerate a random protein removal, but sensitive to the removal of hub proteins (Hao et al., 2016). As a result, our PPI network shows 25 proteins interacting with each other. Among these DEGs, five hub genes in the PPI network were selected using cytoHubba, and these included ALB, EGF, FN1, TTR, and KNG1. The analysis showed that FN1 was upregulated and the other four genes were downregulated in patients with FSGS. We verified the accuracy of the DEGs identified from the microarray data analysis using the FSGS rat model. We selected certain genes that are closely related to the pathogenesis of FSGS for further verification by qRT-PCR, including EGF, FN1, and TTR. As is known to all, ALB encodes the most abundant protein in human blood, and bioinformatics analysis revealed that ALB was downregulated in FSGS, which is consistent with the clinical characteristics of known nephrotic syndrome (Wang and Greenbaum, 2019). FN1 encodes fibronectin, which is involved in a series of biological processes, such as embryogenesis, host defense and transfer, blood coagulation, and wound healing (Waalkes et al., 2010). FN1 expression is significantly upregulated in renal biopsies from patients with diabetic nephropathy compared with its expression in control biopsies (Kliewe et al., 2019). Mutation of the FN1 gene can lead to glomerular disease with fibronectin deposition (GFND) (Castelletti et al., 2008), and the expression of FN1 is increased in renal fibrosis (Stribos et al., 2016). Through qRT-PCR experiments, we verified that FN1 expression was increased in the FSGS group, which was consistent with previous screening results. EGF is an effective mitotic factor that plays a key role in the growth, proliferation and differentiation of a variety of cell types. EGF also plays a vital role in the activation pathway that mediates podocyte damage and loss in diabetic nephropathy, and loss of the podocyte EGF receptor might mitigate diabetic nephropathy (Chen et al., 2015). In patients with IgA nephropathy, both renal EGF expression and urine EGF excretion are reduced at baseline and negatively correlated with disease progression (Stangou et al., 2009). Analogously, urinary EGF levels are reduced in patients with congenital ureteropelvic junction obstruction (Grandaliano et al., 2000). EGF has been shown to enhance the PI3K/Akt pathway (Xie et al., 2018). In addition, the KEGG pathway database showed that EGF can stimulate the FOXO pathway through the MAPK signaling pathway. Through qRT-PCR experiments, we verified that EGF expression was decreased in the FSGS group, which was consistent with previous screening results. TTR encodes the thyroxine transporter protein, which is used to transport thyroid hormones in plasma and cerebrospinal fluid. Mutations in this gene have been linked to amyloid deposition, which mainly affects peripheral nerves or the heart. Some studies have found that TTR might be a potentially valuable target for rhabdomyolysis induction of acute kidney injury (AKI), which suggests that reducing TTR can increase reactive oxygen species production and induce apoptosis (Li et al., 2017). Through qRT-PCR experiments, we verified that TTR expression was decreased in the FSGS group, which was consistent with previous screening results. KNG1 produces two different proteins by selective splicing, namely, high-molecular-weight kininogen (HMWK) and low-molecular-weight kininogen (LMWK). HMWK is needed for blood clotting and assembly of the kinin-kinin system, whereas unlike HMWK, LMWK is not needed for coagulation. Urinary KNG1 is decreased in patients with AKI (Gonzalez et al., 2016). Regrettably, we did not verify the reduction of KNG1 in the FSGS group.

Through ROC curve analysis, we further revealed that EGF, FN1, and TTR may be potential markers for patients with FSGS. Among these three genes, FN1 showed the most accurate diagnostic result with the highest AUC value.

In summary, this study presents a comprehensive bioinformatics analysis of DEGs that are likely linked to the progression of FSGS, and we identified the key genes that are closely related to the initiation and progression of FSGS and the related signaling pathways. These genes can potentially be used for the molecular diagnosis or treatment of FSGS, but the mechanism of action of these genes in FSGS remains to be further investigated.

## Conclusion

In conclusion, the DEGs identified by bioinformatics data analysis combined with experimental validation, including EGF, FN1, and TTR, may potentially be used as targets for FSGS diagnosis and treatment.

## Data Availability

The datasets presented in this study can be found in online repositories. The names of the repository/repositories and accession number(s) can be found in the article/supplementary material.
